# Plasma RANTES, IL-10, and IL-8 levels in non–small-cell lung cancer patients treated with EGFR-TKIs

**DOI:** 10.1186/1756-0500-6-139

**Published:** 2013-04-08

**Authors:** Kanako Umekawa, Tatsuo Kimura, Shinzoh Kudoh, Tomohiro Suzumura, Takako Oka, Misato Nagata, Shigeki Mitsuoka, Kuniomi Matsuura, Toshiyuki Nakai, Naruo Yoshimura, Yukimi Kira, Kazuto Hirata

**Affiliations:** 1 Department of Respiratory Medicine, Graduate School of Medicine, Osaka City University, 1-4-3 Asahi-machi, Abeno-ku, Osaka 545-8585, Japan; 2 Department of Central Laboratory, Graduate School of Medicine, Osaka City University, Osaka, Japan

**Keywords:** NSCLC, EGFR-TKIs, RANTES, IL-8, IL-10

## Abstract

**Background:**

Epidermal growth factor receptor (EGFR) tyrosine kinase inhibitors (TKIs), routinely used to treat advanced non-small-cell lung cancer (NSCLC) patients with activated *EGFR* mutations, are associated with excellent response and improved performance status. Recently, pro-inflammatory cytokines, such as regulated upon activation normal T cell expressed and secreted (RANTES), interleukin (IL)-10 and IL-8 have been proposed as mediators of cancer development. EGFR-TKIs have been found to affect this network of pro-inflammatory cytokines.

**Methods:**

EGFR-TKIs (erlotinib, 150 mg/day; and gefitinib, 250 mg/day) were administered once per day. Treatment was continued until disease progressed or the patient developed intolerable symptoms of toxicity, or withdrew his/her consent for study participation. The treatment was a part of standard care. We investigated the correlation between plasma pro-inflammatory cytokines (including plasma RANTES, IL-10, and IL-8) levels and clinical outcomes following EGFR-TKI treatment in lung cancer patients. Pro-inflammatory cytokine levels were evaluated at diagnosis and on treatment day 30 after the first administration of EGFR-TKIs.

**Results:**

Overall, 33 patients were enrolled. Plasma pro-inflammatory cytokine levels were determined for all patients at diagnosis. Plasma samples from 26 patients were obtained on treatment day 30. High level of RANTES at diagnosis was associated with severe general fatigue (P = .026). Low level of RANTES at diagnosis was significantly associated with long-term survival (P = .0032). Percent decrease change of IL-10 was associated with severity of rash (P = .037). The plasma IL-8 level on treatment day 30 (median, 5.48 pg/mL; range, 0.49–26.13 pg/mL) was significantly lower than the level at diagnosis (median 10.45 pg/mL; 3.04–54.86 pg/mL; P = .021).

**Conclusions:**

These results suggest that EGFR-TKIs may suppress systemic inflammation and promote tumor shrinkage. The network of pro-inflammatory cytokines was affected by EGFR-TKI treatment for NSCLC. In addition, the clinical outcomes of EGFR-TKI treatment were influenced by the status of the plasma pro-inflammatory cytokines at diagnosis.

## Background

Cancer is associated with systemic inflammation driven by multiple pro-inflammatory cytokines [1]. The network of pro-inflammatory cytokines such as regulated upon activation normal T-cell expressed and secreted (RANTES), interleukin (IL)-10, and IL-8 have been proposed as mediators of cancer development [1,2]. Pro-inflammatory cytokines play roles in catabolism, gluconeogenesis, and acute-phase protein production [1]. They also play protective roles during the first stages of inflammation; however, persistent continuation has deleterious effects.

Gefitinib and erlotinib are orally administered epidermal growth factor receptor (EGFR) tyrosine kinase inhibitors (TKIs) used for the treatment of non–small-cell lung cancer (NSCLC) in patients with activated mutations of the *EGFR* gene [3-6]. Unlike treatment with cytotoxic agents, EGFR-TKIs are associated with excellent response rates, prolonged survival, low numbers of adverse hematological events, and improved quality of life. EGFR signaling is triggered by the binding of EGF and EGF-like growth factors, resulting in the homodimerization of EGFR molecules or heterodimerization of EGFR with other closely related receptors such as c-erbB2 [7]. EGF-stimulated EGFR phosphorylation [8] promotes cancer cell proliferation through the downstream phosphoinositide 3-kinase (PI3K)/Akt and extracellular signal-regulated kinase (ERK1/2) pathways [9]. PI3K/Akt and ERK1/2 pathways are activated in lung cancer [10] and are closely associated with cancer cell proliferation [11,12].

RANTES is a known chemotactic cytokine that is produced by many cell types, including T-lymphocytes, monocytes, platelets, eosinophils, epithelial cells, dendritic cells, and mast cells [13]. RANTES, which is transcribed and secreted not only by T cells, other inflammatory cells, and stromal cells, but also tumor cells and nonmalignant bronchial epithelium, is involved in immunoregulatory and inflammatory processes [14]. RANTES has been used as a prognostic indicator in both breast and cervical cancers and high levels of RANTES in these malignancies correlates with a poor outcome [14,15]. RANTES in breast carcinoma is associated with invasion, metastasis, and poor clinical survival [16,17]. Protein kinases C (PKC) α and β have been shown to affect tumor progression and malignant phenotype [18,19]. PKCα plays an obligatory role in EGFR transactivation and signaling to ERK1/2 activation [20-22]. PKCα-dependent EGFR transactivation may contribute to the development and maintenance of the androgen-refractory phenotype in advanced prostate cancer [22]. PKCα/β activator 12-*O*-tetradecanoylphorbol-13-acetate (TPA) only induces IL-8 expression, whereas both inhibit tumor necrosis factor (TNF)-α induced RANTES expression [2].

IL-10, an immunoregulatory component in the cytokine network, is mainly expressed by monocytes, macrophages, T cells, and normal and neoplastic B cells [23]. IL-10 is associated with tumor malignancy via immune escape. IL-10 promotes tumor malignancy by promoting T cell apoptosis and tumor cell survival [24]. Marked decrease in plasma IL-10 levels accompanies marked increase in RANTES levels in patients with severe, treatment-resistant atopic dermatitis [25]. Previous reports have shown that IL-10 has different prognostic significance in early and late stage lung cancer patients [23]. Absence of IL-10 expression is associated with poor outcome in stage I NSCLC, whereas presence of IL-10 positive macrophages in late stage NSCLC is an indicator of poor prognostic outcome. Moreover, persistence of EGFR and IL-10 in the blood of colorectal cancer patients after surgery indicates a high risk of relapse in patients [26].

IL-8 is a cytokine of the CXC chemokine family and acts as a ligand for 2 G-protein coupled receptors [7]. In addition to its role in neutrophil recruitment and activation, IL-8 is thought to be involved in a wide variety of other processes such as angiogenesis and the formation of metastases in lung cancer [27,28]. EGF has been demonstrated to initiate the release of IL-8 from bronchial epithelial and lung cancer cells [9,29,30]. ERK phosphorylation is associated with IL-8 expression in airway epithelium cells [31,32]. An *in vitro* study has shown that the ability of IL-8 to increase cell proliferation is blocked by an inhibitor of EGFR tyrosine kinase [7]. IL-8 is positively regulated by EGFR signaling, whereas EGFR inhibitors block IL-8 expression [33]. In the nude mice model, treatment with monoclonal antibody C225, directed against the EGFR, inhibits mRNA and protein production of IL-8 [34].

EGFR-TKIs are thought to partially affect these cancer related pro-inflammatory cytokine networks. To test this hypothesis, we investigated the correlation between plasma pro-inflammatory cytokine levels and clinical outcomes following EGFR-TKI treatment in lung cancer patients. Pro-inflammatory cytokine levels were evaluated at diagnosis and on treatment day 30 after the first administration of EGFR-TKIs.

## Methods

### Patients

Eligible patients had pathologically confirmed advanced NSCLC that recurred after 1 or 2 prior chemotherapies. Each patient was required to meet the following criteria: adequate organ function, performance status (PS) of 0–2, and no other active malignancies. Mutations in the tyrosine kinase domain (exons 18–21) of *EGFR* were identified using the peptide nucleic acid (PNA) clamp polymerase chain reaction (PCR) assay [35]. Written informed consent was obtained from all patients. This study was approved by the Osaka City University Institutional Review Board (approval number: 1377).

### Treatment, response, and clinical outcome

EGFR-TKIs (erlotinib, 150 mg/day; and gefitinib, 250 mg/day) were administered once per day. Treatment was continued until disease progressed or the patient developed intolerable symptoms of toxicity, or withdrew his/her consent for study participation. The treatment was a part of standard care. The objective responses of each lesion examined were assessed every 4 weeks following commencement of EGFR-TKIs administration by using the Response Evaluation Criteria in Solid Tumors, version 1.0 [36]. Toxicity was graded according to the National Cancer Institute Common Toxicity Criteria Version 3.0 [37]. EGFR-TKIs related non-hematologic toxicities of grade 3 and 4 were managed by reducing the dose of EGFR-TKIs. The progression-free survival (PFS) or overall survival (OS) was calculated from the start of EGFR-TKI treatment to the date of disease progression or death.

### Analyses of plasma pro-inflammatory cytokines

Plasma samples were collected at diagnosis and on treatment day 30. Venous blood (7 mL) was collected in EDTA (anticoagulant)-containing tubes and immediately centrifuged at 3000 rpm for 15 min. Plasma samples were frozen at -80°C until analysis. Plasma RANTES, IL-10, and IL-8 levels were measured using the Luminex 200× PONENT system (Milliplex MAP kit; Millipore, Billerica, MA, USA), according to the manufacturer’s instructions. Plasma RANTES, IL-10, and IL-8 levels were estimated as previously reported [38]. Briefly, 25 μL of plasma was incubated with antibody-linked beads overnight at 4°C, rinsed twice with the washing solution, and incubated for 1 hour with biotinylated secondary antibodies. Data acquisition using the Luminex system was performed after a final incubation with streptavidin-phycoerythrin for 30 minutes. The minimum detectable concentrations of plasma RANTES, IL-10, and IL-8 levels were 69.00, 21.50, and 13.25 pg/mL, respectively. All samples were assayed in duplicate.

### Statistical analyses

All values are expressed as the median and range. Statistical comparisons of pro-inflammatory cytokine levels before and after treatments were performed using the Wilcoxon signed-ranks test. The association between the plasma pro-inflammatory cytokine levels at diagnosis and the effects of EGFR-TKI treatments was assessed using the Mann-Whitney test. The association between the changes of plasma pro-inflammatory cytokine levels and the effects of EGFR-TKI treatments was also assessed with Mann–Whitney test. Stepwise multiple regression analysis was performed to evaluate the independent relationship of overall survival with age, sex, stage IIIb or IV, EGFR mutation status, plasma RANTES, IL-10, and IL-8 levels at diagnosis. Two-tailed P values <0.05 were considered significant. A box plot provides information about the median, variability, and outliers of data distribution. The horizontal line within each box indicates the sample median. The plot consists of a box that extends from the 25th quantile to the 75th quantile. The box lines that extend from each end to the outermost data point that falls within the distances were computed as follows: 1st quartile +1.5* (interquartile range) and 3rd quartile +1.5* (interquartile range). Data points outside these computed ranges were considered outliers. All statistical analyses were carried out using the JMP 8.0 statistical program (SAS Institute, Inc., Cary, NC, USA).

## Results

### Patient characteristics

Thirty-three patients were enrolled between September 2008 and October 2009. Adequate plasma samples could not be obtained for analyses from 7 patients on treatment day 30. *EGFR* mutation status was positive in 19 patients, negative in 9, and unknown in 5. Initially, none of the patients with *EGFR* mutations had the secondary T790M mutation. The numbers of patients with concomitant diseases and drugs, which influence cytokine levels, were as follows: chronic rheumatoid arthritis (1), diabetes mellitus (6), hyperlipidemia (6), and hypertension (17). No patient had an infectious disease. The patient population profile is provided in Table 1.

**Table 1:** Patient characteristics (n = 33)

**Characteristics**	**Number**	
Age, years		
Median (range)	67	(54–86)
Sex		
Male/female	18/15	
Performance status		
0/1/2	2/28/3	
Clinical stage		
III/IV	6/27	
Histology		
Ad/Sq/La	29/3/1	
*EGFR* mutation status		
Ex18 del/ Ex19 del/ Ex21L858R	1/9/9	
Negative	9	
Unknown	5	
Brinkman index		
Median (range)	370	(0–3840)
Body mass index, Kg/m^2^		
Median (range)	23.0	(16.0–29.8)
Concomitant disease		
rheumatoid arthritis	1	
diabetes mellitus	6	
hyperlipidemia	6	
hypertension	17	
Treatment line		
1st/2nd/more	3/19/11	

*EGFR*, Epidermal growth factor receptor; *Ad, *Adenocarcinoma; *Sq,* Squamous cell carcinoma, *La, *Large-cell carcinoma; *Ex, *Exon; *del*, Deletion.

### Clinical outcomes

The rate of rash, diarrhea, appetite loss, general fatigue, and liver dysfunction of all grades was 97.0%, 30.3%, 48.5%, 50.0%, and 38.5%, respectively. Response to EGFR-TKI treatments included partial response (PR) in 8 cases (24%), stable disease (SD) in 14 (42%), and progressive disease (PD) in 11 (33%). Patients in all of 8 PR cases, 8 of 14 SD cases, and 3 of 11 PD cases showed *EGFR* mutations. On treatment day 30, treatment was stopped for 7 of 33 patients because of PD (4 patients) and side effects (3 patients: grade 3 drug-induced pneumonitis in 1 patient and grade 3 diarrhea in the other 2 patients). The median PFS and OS were 102 days (6–1442 days) and 255 days (70–1447 days), respectively.

### Clinical features associated with pro-inflammatory cytokine levels

**Figure 1:**
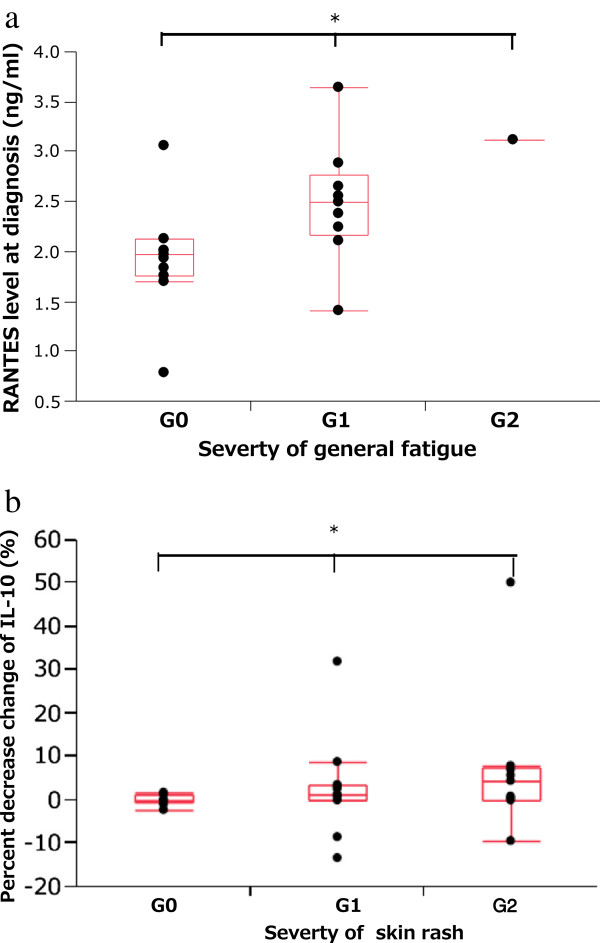
**Association of plasma interleukin (IL)-10 and regulated upon activation normal T cell expressed and secreted (RANTES) levels with epidermal growth factor receptor (EGFR) tyrosine kinase inhibitor (TKI) toxicity. **(**a**) Plasma RANTES levels at diagnosis show significant correlation with the severity of general fatigue (P = .025). (**b**) Percent decrease change of IL-10 was associated with severity of rash (P = .037).

First, we analyzed the association between pro-inflammatory cytokine levels at diagnosis and patient characteristics (Table 2). High levels of plasma IL-8 at diagnosis showed significant positive associations with the Brinkman index (P = .0063). No significant associations were observed between plasma IL-10 or RANTES levels and other patient characteristics at diagnosis. Second, we analyzed the association between pro-inflammatory cytokine levels at diagnosis and adverse effects observed following EGFR-TKI treatment. High level of plasma RANTES at diagnosis was associated with the severity of general fatigue (P = .025, Figure 1a). Percent decrease change of plasma IL-10 was associated with severity of rash (P = .037, Figure 1b). Third, we analyzed the association between pro-inflammatory cytokine levels at diagnosis and the clinical efficacy of the EGFR-TKI treatment. *EGFR* mutations, sex, and low level of plasma RANTES at diagnosis were significantly associated with long-term survival (P = .0044, .037, and .046, respectively). In a multivariate logistic regression model, *EGFR* mutations, sex, and low level of plasma RANTES at diagnosis were identified as significantly positive prognostic factors (R^2^ = .26, P = .004; R^2^ = .43, P = .037; R^2^ = .52, P = .045, respectively) (Table 3). No significant associations were observed between plasma pro-inflammatory cytokine levels at diagnosis and treatment responses.

**Table 2:** Summary of plasma pro-inflammatory cytokines levels at diagnosis in patients with NSCLC

**Case**	**Histrogical type**	***EGFR *mutation status**	**Brinkman index**	**Treatment agent**	**Response**	**IL-8 (ng/mL)**	**IL-10 (ng/mL)**	**RANTES (pg/ml)**
**1**	Ad	Ex21 L858R	900	gefitinib	PR	5.79	1.67	2
**2**	Ad	Ex21 L858R	0	erlotinib	PR	3.33	94.2	1.41
**3**	Ad	Ex19 del	0	erlotinib	PR	4.09	126	2.13
**4**	Ad	Ex21 L858R	0	gefitinib	PR	3.92	18.1	1.85
**5**	Ad	Ex21 L858R + Ex19 del	370	erlotinib	PR	21.9	1.23	2.11
**6**	Ad	Ex19 del	0	erlotinib	PR	7.05	1.37	2.56
**7**	Ad	Ex19 del	0	gefitinib	PR	4.82	1.59	2.02
**8**	Ad	Ex21 L858R	1640	gefitinib	PR	54.9	2.7	2.38
**9**	Ad	negative	400	erlotinib	SD	34.9	3.39	3.66
**10**	Ad	negative	0	erlotinib	SD	27.1	4.94	2.25
**11**	Ad	unknown	1800	erlotinib	SD	NE	NE	NE
**12**	Sq	Ex19 del	3840	gefitinib	SD	12.6	1.1	1.98
**13**	Ad	Ex19 del	30	erlotinib	SD	5.27	0.69	2.66
**14**	Ad	Ex19 del	0	erlotinib	SD	10.5	0.85	2.5
**15**	Ad	unknown	300	erlotinib	SD	7.69	2.61	1.7
**16**	La	negative	2080	erlotinib	SD	NE	NE	NE
**17**	Sq	Ex21 L858R	750	erlotinib	SD	11.6	56.3	3.06
**18**	Ad	unknown	600	erlotinib	SD	13.7	1.1	2.82
**19**	Ad	Ex21 L858R	0	erlotinib	SD	20.6	11.6	0.783
**20**	Ad	Ex19 del	0	erlotinib	SD	14.8	2.07	2.02
**21**	Ad	Ex21 L858R + Ex19 del	0	gefitinib	SD	3.04	1.3	1.76
**22**	Ad	Ex19 del	200	gefitinib	SD	10.9	1.59	1.91
**23**	Sq	unknown	1560	erlotinib	PD	NE	NE	NE
**24**	Ad	negative	1080	erlotinib	PD	25.2	8.15	1.57
**25**	Ad	unknown	0	erlotinib	PD	NE	NE	NE
**26**	Ad	Ex18 G719A	2000	erlotinib	PD	11.6	4.48	3.12
**27**	Ad	unknown	1410	erlotinib	PD	NE	NE	NE
**28**	Ad	Ex19 del	1000	gefitinib	PD	9.44	2.25	2.89
**29**	Ad	negative	1100	erlotinib	PD	56.3	5.63	2.16
**30**	Ad	negative	2000	erlotinib	PD	15.6	0.85	0.292
**31**	Ad	negative	825	erlotinib	PD	8.27	1.59	2.13
**32**	Ad	Ex21 L858R	0	erlotinib	PD	5.34	7.05	1.7
**33**	Ad	negative	1680	erlotinib	PD	57.7	1.63	2.06

*NSCLC, *Non-small-cell lung cancer; *Ad, *Adenocarcinoma; *Sq,* Squamous cell carcinoma; *La, *Large-cell carcinoma; *Ex, *Exon; *del*, Deletion; *PR, *Partial response; *SD, *stable disease; *PD, *Progression disease; *IL-8, *Interleukin-8; *IL-10,* Interleukin-10; *RANTES, *Regulated upon activation normal T-cell expressed and secreted.

**Table 3:** Step-wise multiple regression analysis between overall survival and clinical parameters

**Variable**	**Standardized regression coefficient**	***F***	**P value**	**Change in *R***^**2**^
Age	-	0.042	0.840	0.604
Sex	-108.81	4.883	0.037	0.431
*EGFR *mutation status	-164.64	9.954	0.004	0.258
Stage	-	1.093	0.307	0.604
IL-8 level at diagnosis	-	0.001	0.973	0.604
IL-10 level at diagnosis	3.30	3.726	0.066	0.584
RANTES level at diagnosis	-0.15	4.453	0.045	0.517

*EGFR,* Epidermal growth factor receptor; *IL-8, *Interleukin-8; *IL-10, *Interleukin-10; *RANTES, *Regulated upon activation normal T-cell expressed and secreted.

### Plasma pro-inflammatory cytokine levels before and after EGFR-TKI treatment

**Figure 2:**
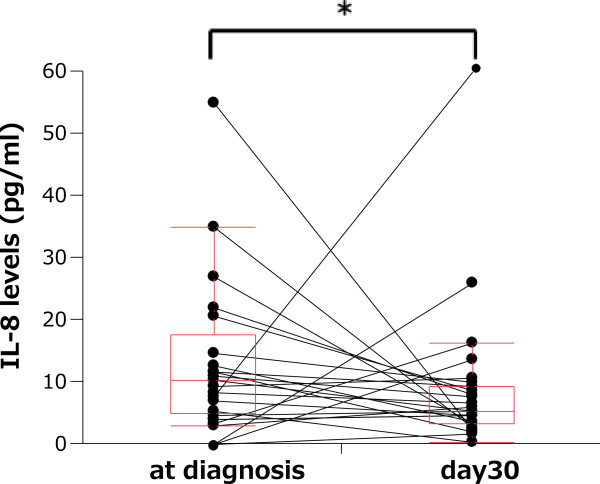
**Comparison of the plasma interleukin (IL)-8 levels before and after epidermal growth factor receptor (EGFR) tyrosine kinase inhibitor (TKI) treatment. **The IL-8 (5.48 pg/mL; range, 0.49–26.13 pg/mL) levels on treatment day 30 were significantly lower than levels at diagnosis (10.45 pg/mL; range, 3.04–54.86 pg/mL; P = .021).

To examine the effect of EGFR-TKI treatment on the network of pro-inflammatory cytokines, we analyzed pro-inflammatory cytokine levels in the 26 patients still receiving treatment on day 30. The plasma IL-8 (5.48 pg/mL; range, 0.49–26.13 pg/mL) level on treatment day 30 was significantly lower than the level at diagnosis (10.45 pg/mL; range, 3.04–54.86 pg/mL; P = .021) (Figure 2). The plasma levels of other pro-inflammatory cytokines at diagnosis, including IL-10 (2.16 pg/mL; range 0.69–125.8 pg/mL) and RANTES (2.08 ng/mL; range 0.29–3.66 ng/mL), showed no significant change on treatment day 30.

## Discussion

We demonstrated that pro-inflammatory cytokines were affected by EGFR-TKI treatment for NSCLC. High level of plasma RANTES at diagnosis was associated with the severity of general fatigue. Low level of plasma RANTES at diagnosis was significantly associated with long-term survival by univariate and multivariate analyses. Percent decrease change of plasma IL-10 was associated with the severity of rash. Decreased level of plasma IL-8 was observed after EGFR-TKI treatment.

Tumor-derived RANTES has been detected in many clinical specimens [15]. In our study, high level of plasma RANTES at diagnosis was associated with the severity of general fatigue. Low level of plasma RANTES at diagnosis was significantly associated with long-term survival. Thus, patients with high systemic inflammation, as represented by RANTES, may experience severe general fatigue and shorter survival time. Moran *et al*. found a correlation between increased RANTES expression and tumor lymphocytic response in lung cancer patients [17]. However, elevated RANTES expression correlated with improved survival in patients with early stage NSCLC [16]. The clinical stage of our patients was advanced, with 6 patients showing stage III and 27 showing stage IV. This may explain the completely different results of Moran *et al*. [17].

The determinants of tumor response and survival were assessed in patients treated with EGFR-TKIs. The multivariate Cox proportional hazards model showed that time since diagnosis and good performance status were significant predictors of survival, and survival correlated with the occurrence and severity of rash [6]. Other reports show that mutations in the *EGFR* are predictive and prognostic indicators in patients with NSCLC treated with erlotinib [39] and gefitinib [40]. In our study, the significant prognosis factors in the multivariate analysis were *EGFR* mutation status, sex, and plasma RANTES, not PS. Patient eligibility in this study required a threshold criteria of PS 0/1. Therefore, the small number of PS 2 may be the reason why PS was not a significant prognostic factor in the multivariate analysis.

Skin toxicity is the most frequently encountered toxicity in patients treated with EGFR-TKIs, and it is believed to result from direct interference of the drug function and EGFR signaling in the skin [41]. EGFR is expressed in the basal layer of the epidermis. Roles of EGFR include stimulation of epidermal growth, inhibition of differentiation, and acceleration of wound healing [41]. Inhibition of mitogen activated protein kinase (MEK), a downstream effector in the EGFR pathway, also leads to papulopustules, suggesting a mechanism-based effect. Similar inflammatory events may also account for periungual inflammation and onycholysis, whereas abnormalities in keratinocyte differentiation may explain impaired stratum corneum leading to xerosis and pruritus [42]. A recent report showed that the macrophage inflammatory protein (MIP)-1β levels are significantly lower in patients with skin toxicity compared to the levels in patients with no skin toxicity [43]. In atopic dermatitis, a marked increase in plasma RANTES levels accompanied by a marked decrease in IL-10 levels is observed [25]. Suppression of Th1 cells by Th2 cells seems to be abrogated by decreased IL-10 and Th2 cytokines, which may be mediated through elevated RANTES in patients with severe atopic dermatitis. In our study, percent decrease change of plasma IL-10 was associated with the severity of rash. Therefore, immune responses mediated by MIP-1β and plasma IL-10 may play a role in the healing process of keratinocytes damaged by EGFR-TKIs.

In our study, EGFR-TKI treatment suppressed tumor proliferation and improved PS and quality of life. At the molecular level, EGFR inhibitors suppress EGFR phosphorylation and inhibit the downstream signals of PKC and ERK, which are associated with IL-8. As a result, EGFR-TKI treatment decreased plasma IL-8 levels. We previously reported that increased adiponectin and decreased insulin levels are observed after EGFR-TKI treatment [44]. This circumstance may improve cancer related anorexia. Our 2 results suggest that EGFR-TKIs may improve cancer cachexia as a consequence of tumor shrinkage and suppress cancer related systemic inflammation.

Our study has certain limitations. The number of patients enrolled was small, and we did not evaluate the differences between the effects of cytotoxic agents and EGFR-TKIs on pro-inflammatory cytokines. The relationship between the concentrations of pro-inflammatory cytokines and tissue immunoreactivity remains to be elucidated.

## Conclusion

High level of plasma RANTES at diagnosis was associated with the severity of general fatigue. Low level of plasma RANTES at diagnosis was significantly associated with long-term survival by univariate and multivariate analyses. Percent decrease change of plasma IL-10 level was associated with the severity of rash. Decreased plasma IL-8 level was observed after EGFR-TKI treatment. The network of pro-inflammatory cytokines was affected by EGFR-TKI treatment for NSCLC. In addition, the clinical outcomes of EGFR-TKI treatment were influenced by the status of the plasma pro-inflammatory cytokines at diagnosis. Our study may provide useful information regarding patient outcomes after EGFR-TKI treatment. A large clinical trial is required to clarify these results.

## Abbreviations

IL-8: Interleukin-8; IL-10: Interleukin-10; RANTES: Regulated upon activation normal T cell expressed and secreted; EGFR-TKIs: Epidermal growth factor receptor-tyrosine kinase inhibitors; NSCLC: Non-small cell lung cancer; PKC: Protein kinases C; ERK1/2: Extracellularly related kinase 1/2; PI3K: Phosphoinositide 3-kinase; PS: Performance status; PFS: Progression free survival; OS: Overall survival; PR: Partial response; SD: Stable disease; PD: Progressive disease; MEK: Mitogen activated protein kinase; SD: Standard deviation; BMI: Body mass index; Ex: Exon; del: Deletion; Ad: Adenocarcinoma; Sq: Squamous cell carcinoma; La: Large cell carcinoma; MIP: Macrophage inflammatory proteins.

## Competing interests

The authors declare that they have no competing interests.

## Authors’ information

KU, TS, TO, MN, and TN are MDs and students at the Graduate School of Medicine, Osaka City University. TK, SM, KM, and NY are MD, PhD and assistant professors at the Graduate School of Medicine, Osaka City University. SK is an MD, PhD and hospital professor at the Graduate School of Medicine, Osaka City University. YK is a PhD and technical staff member in the Department of Central Laboratory, Graduate School of Medicine, Osaka City University. KH is an MD, PhD, and professor at the Graduate School of Medicine, Osaka City University.
